# Preparation and Properties of PED-TDI Polyurethane-Modified Silicone Coatings

**DOI:** 10.3390/polym14153212

**Published:** 2022-08-06

**Authors:** Xiaojun Xi, Zhanping Zhang, Yuhong Qi

**Affiliations:** Department of Materials Science and Engineering, Dalian Maritime University, Dalian 116026, China

**Keywords:** antifouling coating, silicone, polyurethane modification, composition of the soft segment

## Abstract

To explore the influence mechanisms of polyurethane soft segments on modified silicone coatings, a series of modified coatings was prepared by introducing different contents of hydroxypropyl-terminated polydimethylsiloxane (PDMS2200) into the soft segment of polyurethane. ATR-FTIR, NMR, CLSM, AFM, contact angle measurement, the tensile test, bacterial adhesion, and the benthic diatom adhesion test were used to investigate the structure, morphology, roughness, degree of microphase separation, surface energy, tensile properties, and antifouling properties of the modified coatings. The results show that PDMS2200 could aggravate the microphase separation of the modified coatings, increase the surface-free energy, and reduce its elastic modulus; when the microphase separation exceeded a certain degree, increasing PDMS2200 would decrease the tensile properties. The PED-TDI polyurethane-modified silicone coating prepared with the formula of PU-Si17 had the best tensile properties and antifouling properties among all modified coatings.

## 1. Introduction

Marine biofouling is a serious problem that ships cannot avoid, which is mainly caused by the adsorption and growth of marine organisms, such as bacteria and diatoms, on the hull’s surface [1,2]. The existence of these organisms will not only increase the roughness of the hull, thereby increasing the fuel consumption and limiting the speed of the ship, but also facilitate biological invasion [3,4].

Among all antifouling methods, fouling-release antifouling coatings that can achieve excellent antifouling effects without biocides have great advantages [5,6,7]. They prevent the adhesion of fouling organisms by providing a smooth surface with low friction and low surface-free energy. Even if adsorption is successful, the adhesion between the organisms and the coating is so weak that the fouling could be easily removed by the water stress or mechanical cleaning.

Currently, fouling-release antifouling coatings include organic fluorine coatings and silicone coatings. The latter have lower marine organism adhesion strength and cost [8]. What is more, silicone coatings also have great chemical stability and low glass transition temperature, making them widely used in ship antifouling. However, silicone coatings also have some shortcomings, such as low mechanical strength [9,10], poor adhesion to the substrate [11], and poor static antifouling effects [5,12]. Therefore, they need to be modified to meet the needs during the actual use process.

Common modified materials mainly include alkyd resin, epoxy resin, acrylic resin, and polyurethane [13,14,15,16]. With its good mechanical properties and adhesion, polyurethane can well improve the defects of silicone coatings [17], so it is an excellent modified material. Furthermore, there are two modification methods: physical blending, and chemical modification. The former is easy to operate; yet some segments will be broken before stretching due to the poor compatibility between silicone and polyurethane [18]. Therefore, it is difficult to apply in practical applications; the preparation of the latter is cumbersome, and the modification treatment is mainly achieved by the reaction of isocyanate groups with reactive hydroxyl groups on the end or side chain of silicone [19].

According to the positions and numbers of active hydroxyl groups on the organosilicon segment, the modified products can be divided into block polymers, graft polymers, and interpenetrating network polymers [20]. Dai et al. [21] prepared a new type of organosilicon block copolymer to replace polyether or polyester as the soft segment of polyurethane. The experimental results showed that the mechanical properties of the modified product could be close to pure polyurethane. Barletta et al. [22] introduced hydrophilic and hydrophobic groups into polyurethane in the form of side chains and then prepared modified coatings by reacting polysiloxane with highly reactive hydroxyl groups with polyurethane. This synthesis method can significantly shorten the modified coatings’ preparation process and give the product good amphiphilic and antifouling effects.

However, due to the differences between the soft and hard segments of the polyurethane, the microphase separation of the modified coatings is inevitable. The synthesis method of polyurethane, the composition and chain length of the soft segment [23,24], the composition of the hard segment [25], and even the choice of solvent will affect the degree of microphase separation and ultimately affect the performance of the modified coatings. Wang et al. [26] introduced zwitterions into the polyurethane’s hard segment to improve the microphase separation of the product. The test results showed that the tensile properties of the product were significantly improved, and the glass transition temperature was reduced. Zhang et al. [27] introduced polysiloxane-containing quaternary ammonium salt (QAS) groups into the polyurethane coating, and the results showed that the QAS chain linked by high polar covalent bonds had good compatibility with the polyurethane’s hard segment. Therefore, the migration of hydrophobic polysiloxane segments to the surface of the coating is limited, thus improving the hydrophilicity of the coating.

In this paper, hydroxypropyl polydimethylsiloxane (PDMS2200) with good mechanical properties was used to prepare silicone-modified polyurethane, and a series of modified coatings with different soft segments was prepared by changing the content of PDMS2200 to further explore the effect of polyurethane’s soft segments on the structure and properties of modified coatings. The antifouling mechanism was analyzed by testing the surface and interface properties, tensile properties, and antifouling properties of the modified coatings. It was hoped that a polyurethane-modified silicone coating would be developed that at the same time had good mechanical and antifouling properties.

## 2. Experiment

### 2.1. Materials

Polyether diol (PED, CP) and polyether triol (PET, CP) were purchased from Jiangsu Hai’an Petrochemical Plant (Hai’an, China). Tolylene 2,4-diisocyanate (TDI, AR) and 1,4-butanediol (BDO, AR) were purchased from Shanghai Aladdin Biochemical Technology Co., Ltd. (Shanghai, China). Hydroxypropyl polydimethylsiloxane (PDMS2200, CP), with an average molecular weight of 2000, was purchased from Ark (Fogang) Chemical Materials Co., Ltd. (Qingyuan, China). Dimethyl benzene (AR) was purchased from Fuchen (Tianjin) Chemical Reagent Co., Ltd. (Tianjin, China). Butyl acetate (AR) was purchased from Tianjin Kemiou Chemical Reagent Co., Ltd. (Tianjin, China). Cyclohexanone (AR) and ethyl orthosilicate (AR) were purchased from Tianjin Damao Chemical Reagent Co., Ltd. (Tianjin, China). Hydroxy-terminated polydimethylsiloxane (DY-107, CP) was purchased from Shandong Dayi Chemical Co., Ltd. (Shandong, China). PED, PET, and PDMS2200 were used after negative pressure dehydration for 2.5 h at 110 °C and −0.095 MPa. Dimethyl benzene, butyl acetate, cyclohexanone, and BDO were dehydrated with a 4A molecular sieve.

### 2.2. Synthesis Principle and Preparation Process

To obtain isocyanate-terminated polyurethanes, the molar ratio of isocyanate to hydroxyl R (R = n(NCO)/n(OH)) was controlled at 1.35. That is, the molar ratio of raw materials of the soft segment to the hard segment (composed of 70 mol% TDI and 30 mol% BDO) was 1:4.5. A series of polyurethanes was prepared by gradually increasing the molar percentage of PDMS2200 (X_PDMS_) in the soft segment. The mole percentages of the soft segment raw materials are shown in Table 1.

#### 2.2.1. Synthesis Principle of the Polyurethane

**Figure 1:**
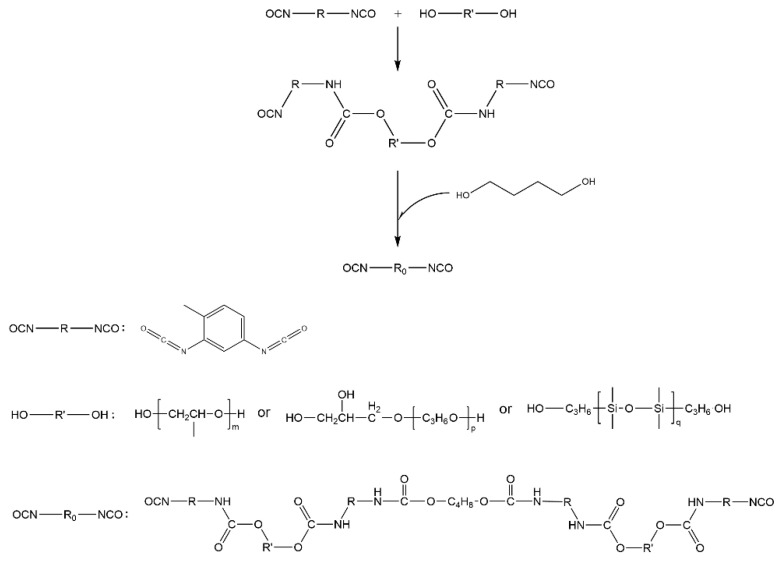
Synthesis principle of PED-TDI polyurethane.

Silicone-modified polyurethane prepolymers were prepared by selecting PED, PET, PDMS2200, and TDI as reaction monomers. After using BDO to extend the chain, the isocyanate-terminated PED-TDI polyurethane was obtained. The reaction principle is shown in Figure 1.

#### 2.2.2. Synthesis Principle of Modified Organic Silicone Resin

**Figure 2:**
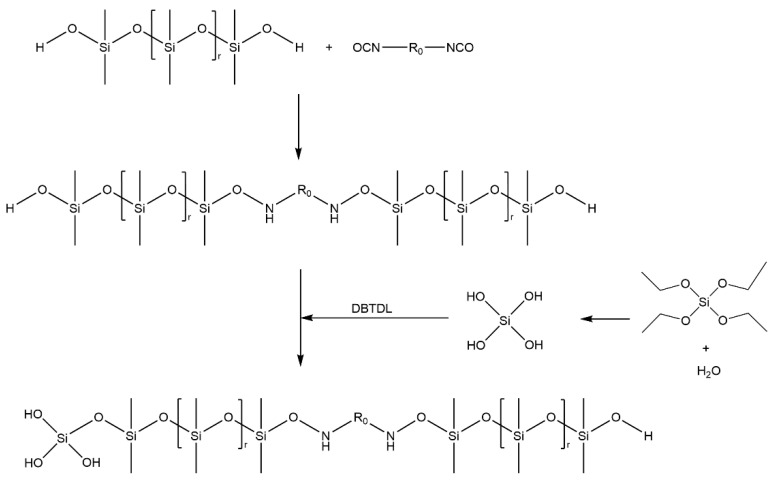
Principle of cross-linking and curing reaction of modified silicone coating.

A polyurethane-modified silicone coating that can be cross-linked and cured at room temperature was prepared in the ratio of DY-107:silicone-modified polyurethane:ethyl orthosilicate:catalyst = 20:5:4:0.5. The reaction principle involved is shown in Figure 2.

#### 2.2.3. Preparation Process

**Figure 3:**
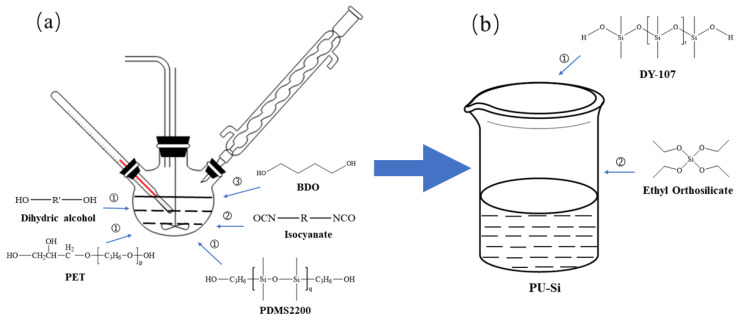
Preparation process of PED-TDI polyurethane-modified silicone coating: (**a**) preparation process of PED-TDI polyurethane; (**b**) preparation process of modified silicone coating.

By referring to the relevant literature [28], the reaction scheme was determined as follows:

Firstly, a four-neck flask was connected to the SZCL-2A digital intelligent control magnetic stirrer (Yuhua Instrument Co., Ltd., Gongyi, China). At the same time, polyol, PDMS2200, and 60 g mixed solvent (consisting of 40 wt.% dimethyl benzene, 40 wt.% butyl acetate, and 20 wt.% cyclohexanone) were added at the proportion shown in Table 1. Under the protection of N_2_, the rotating speed of the magnetic stirrer was controlled to be 160 r/min, and the reaction system was heated to 50 °C by using a DF-101 constant temperature heating magnetic stirrer (China Gongyi Yuhua Instrument Co., Ltd.), and then TDI was added dropwise. After stirring for 30 min, the reaction system was heated to 80 °C. After reacting for 2 h, the reaction system was air-cooled to 50 °C, and 2.6 g BDO was added. After that, the system was heated to 75 °C for a 4 h chain expansion reaction, and polyurethane prepolymer was finally obtained. The specific device is shown in Figure 3a.

PED-TDI polyurethane was added to DY-107 in the proportion shown above, and ethyl orthosilicate and catalyst were successively added. The modified silicone coating was obtained after 20 min of reaction. The process is shown in Figure 3b. The modified products were coated in glass slides and PTFE molds, respectively, and a series of glass slides and mold samples were obtained by crosslinking curing at room temperature. The former was used for structural characterization, surface and interface performance testing, antifouling performance testing, etc. The latter was used for tensile performance testing.

### 2.3. Characterizations

#### 2.3.1. Chemical Structure

Attenuated total reflectance Fourier transform infrared spectroscopy (ATR-FTIR, Thermo Fisher Scientific Inc., Waltham, MA, USA) was used to determine functional groups of polyurethane and modified silicone coatings. The scanning range was 4000–650 cm^−1^, the resolution was 2 cm^−1^, and the number of scans was 32 times.

Nuclear magnetic resonance spectrometer (NMR, Bruker 400M, Bruker Physik-Ag, Karlsruhe, Germany) was used to determine the hydrogen spectra of polyurethane prepolymers and modified organosilicon coatings. The resonance frequency was 400 MHz, and the solvent was CDCl_3_.

#### 2.3.2. Surface Observation

Confocal laser scanning microscopy (CLSM, OLS4000, Olympus, Tokyo, Japan) was used to observe the surface of the modified silicone coating. The roughness was calculated by the LEXT analysis software.

Atomic force microscopy (AFM, Bruker Dimension Icon, Bruker Physik-AG, Karlsruhe, Germany) was used to observe the microphase separation on the surface of modified coatings. The probe type was OTESPA-R3, and the scanning mode was Tapping. The scanning frequency was 1 Hz, and the scanning pixel was 256.

A JC2000C contact angle measurement instrument (Zhongchen Digital Technology Equipment Co., Ltd., Shanghai, China) was used to measure the modified coatings’ water and diiodomethane contact angle, and the surface energy *γ* of the modified coatings was calculated by Owens’s two-liquid method [29].

#### 2.3.3. Tensile Test

A UTM 5105 computer-controlled electronic universal testing machine (Jinan Wance Electrical Equipment Co., Ltd., Jinan, China) was used to test the casting sample at a tensile rate of 50 mm/min, and the elastic modulus, elongation, and fracture strength were calculated according to the stress–strain curves.

#### 2.3.4. Antifouling Evaluation

Bacterial adhesion and benthic diatom tests were carried out on the modified coatings. The antifouling properties of the modified coatings were measured by comparing the bacterial adhesion rate and chlorophyll content on the coating surface. The specific steps are shown in the literature [30,31].

## 3. Results

### 3.1. Chemical Structure

**Figure 4:**
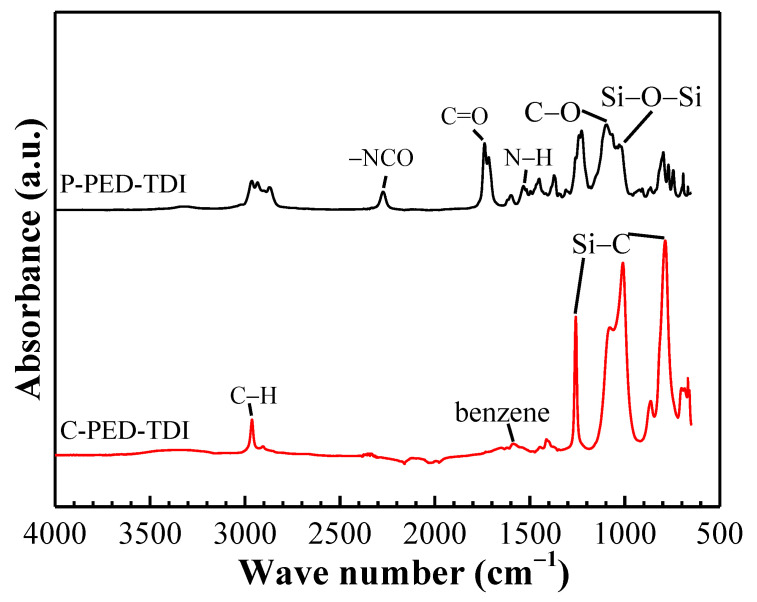
ATR-FTIR spectra of PED-TDI polyurethane and its modified coatings.

The preparation of the modified coatings mainly involves the reaction between hydroxyl and isocyanate. To verify whether the modification is successful, the polyurethane (denoted by P) and its modified coatings (denoted by C) were tested by ATR-FTIR. The results are shown in Figure 4.

For the polyurethane, the characteristic peaks of stretching vibration of N–H, –NCO, C–O, and C=O groups were observed at 1538 cm^−1^, 2270 cm^−1^, 1170 cm^−1^, and 1727 cm^−1^, respectively. These peaks indicated the presence of carbamate in polyurethane. The characteristic peaks at 2970 cm^−1^ (the C–H bond in the Si–CH_3_ group), 1259 cm^−1^ (Si–C bending), 801 cm^−1^ (Si–C vibration), and 1017 cm^−1^ (Si–O–Si stretching) indicated the successful introduction of PDMS2200.

For the modified coatings, the characteristic peaks corresponding to the –NCO groups disappeared, indicating that the free isocyanate groups in the polyurethane have been wholly consumed. At the same time, the characteristic peaks corresponding to Si–CH_3_ groups and Si–O–Si groups in the modified coatings were significantly enhanced, which was caused by the addition of a large amount of silicone resin in the subsequent experiments.

**Figure 5:**
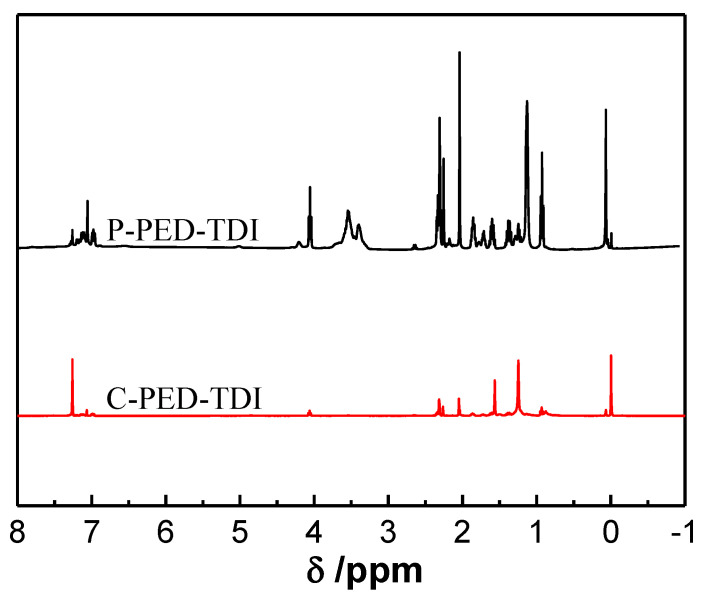
NMR spectra of PED-TDI polyurethane and its modified coatings.

To further analyze the chemical structure of the polyurethane and the modified coatings, their NMR spectra were tested, and the results are shown in Figure 5.

The chemical shift near 0 ppm corresponded to the proton on the Si–CH_3_ group. The chemical shift at δ = 0.79–1.48 ppm was attributed to hydrogen on methylene groups linked to Si in PDMS2200. The chemical shift around 1.7 ppm corresponded to the methyl group on TDI. The chemical shift at δ = 2.0–2.5 ppm was attributed to methyl and methylene groups on PED and PET. The chemical shift at δ = 3.5 ppm corresponded to hydrogen on methyne linked to carbamate; the multiple signal peaks near 4.0 ppm were attributed to the protons in methylene on PDMS2200. Combined with ATR-FTIR spectra, it could be determined that polyurethane-modified silicone coatings were successfully prepared.

### 3.2. Surface Morphology and Roughness

**Figure 6:**
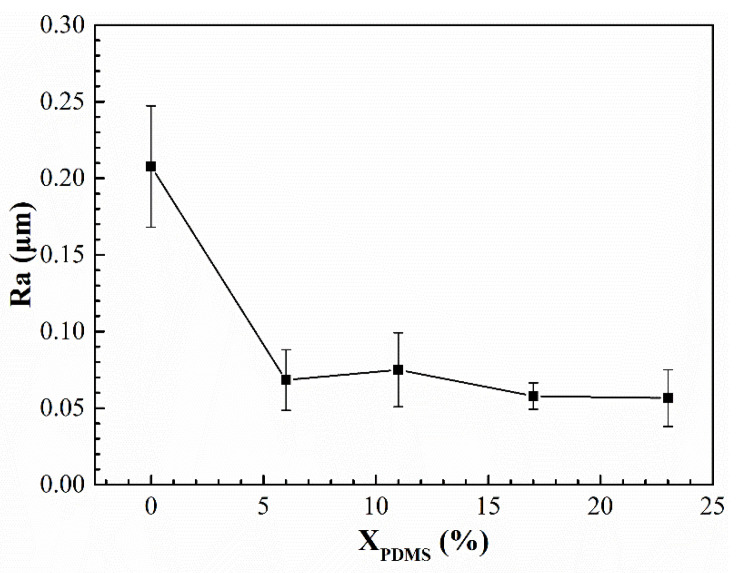
Roughness of modified silicone coatings.

Figure 6 shows the surface roughness of the modified silicone coatings. It was found that with the addition of PDMS2200, the surface roughness of the coatings was greatly reduced. This is because the addition of silicone segments increased the degree of microphase separation of the polyurethane. Compared with the interior of the coating, silicone tended to enrich on the surface. A large amount of silicone formed a continuous phase on the coating’s surface, thereby improving its flatness. With continued increases in X_PDMS_, the continuous phase did not change much, so its roughness was maintained in a relatively stable state.

**Figure 7:**
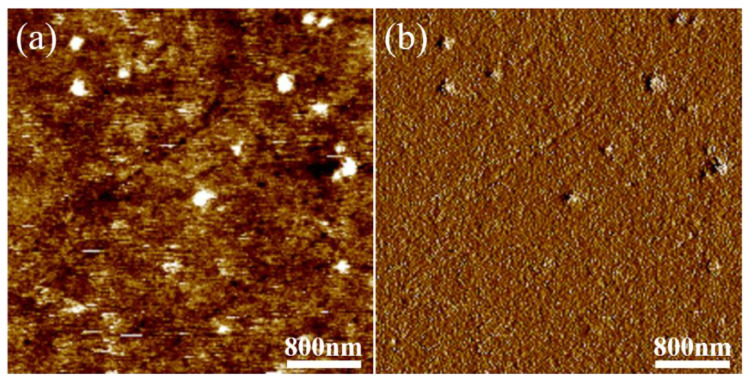
AFM maps of PED-TDI polyurethane-modified silicone coating: (**a**) height maps; (**b**) phase maps.

The microphase separation on the surface of the PU-Si17 coating was observed by AFM, and the results are shown in Figure 7. It was found that apparent phase separation occurred on the surface of the modified coating. The part with a darker color and concave surface corresponds to the soft segment in the coating, and the part with a brighter color and convex surface is the hard segment area. It was seen that the continuous phase on the surface of the coating was composed of soft segments dominated by silicone, and a small number of hard segments was dispersed in the soft segments. This result proves the above inference.

### 3.3. Contact Angle and Surface Free Energy

**Figure 8:**
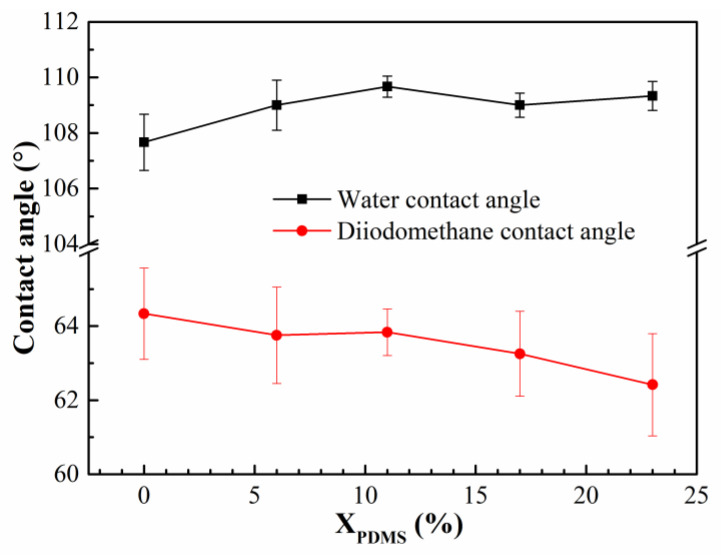
Contact angle of modified silicone coatings.

The contact angles of the modified coatings are shown in Figure 8. All the water contact angles were greater than 107°, indicating that all the modified coatings had good hydrophobicity. Because the addition of PDMS2200 made more silicone enrich on the coatings’ surfaces, improving the hydrophobicity, the water contact angle increased; the decrease of the contact angle may have been caused by the change of its surface roughness.

**Figure 9:**
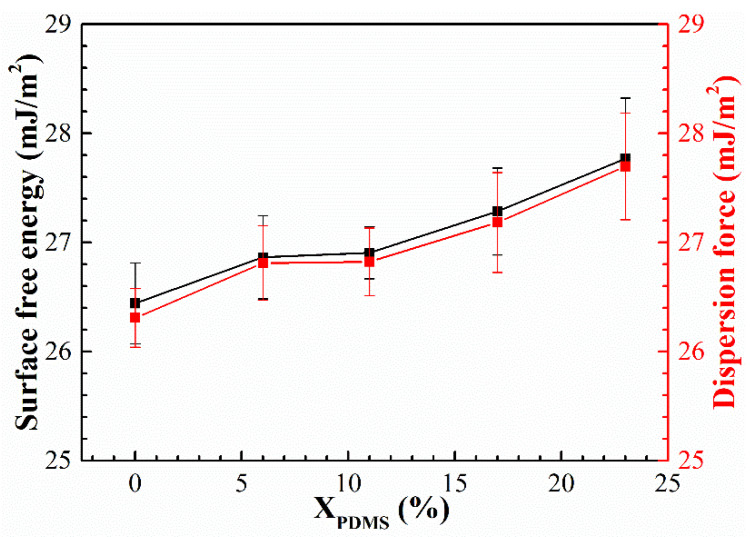
Surface-free energy and dispersion force of modified silicone coatings.

The surface-free energy of the modified coatings was calculated, and the results are shown in Figure 9. It was found that with the increase of PDMS2200, the surface energy of the coatings increased continuously. On the one hand, according to the Cassie model [32,33], the higher the roughness of the coating surface, the more difficult it is for the liquid to wet the surface, and the lower the surface energy; on the other hand, according to the calculation formula of the surface energy [32], it was seen that the surface energy of the coating was composed of the polar force and the dispersion force. Comparing these two forces, it was found that the polar force could be ignored, and the dispersion force was mainly related to the degree of molecular deformation—the greater the degree, the stronger the dispersion force. As shown in Figure 9, with the continuous addition of PDMS2200, a large number of Si and O atoms significantly improved the flexibility of the molecular chain of the modified coatings, thereby increasing the dispersion force of the coating to a certain extent. Furthermore, the surface energy of the coatings continued to increase.

Under the combined effect of roughness and dispersion force, the surface free energy of the modified coatings showed a rising trend with the increase of X_PDMS_.

### 3.4. Tensile Properties

**Figure 10:**
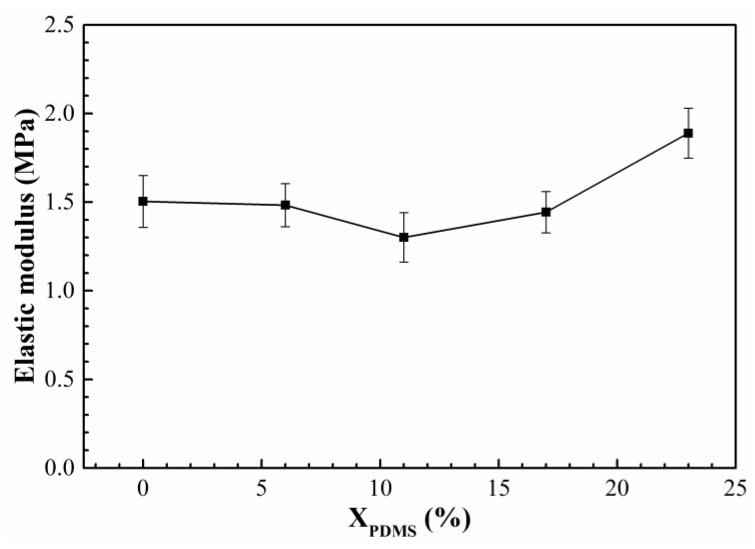
Elastic moduli of modified silicone coatings.

Tensile tests were carried out on the modified silicone coatings, and their elastic modulus was calculated. As shown in Figure 10, the introduction of PDMS2200 reduced the modified coatings’ elastic moduli. This is because PDMS2200 increased the length of the soft segment and improved the flexibility of the molecular chain, so the elastic moduli of the coatings decreased; with the continuous improvement of X_PDMS_, the strengthening effect of the hard segment on the coatings was reflected, thereby improving the elastic moduli of the coatings.

**Figure 11:**
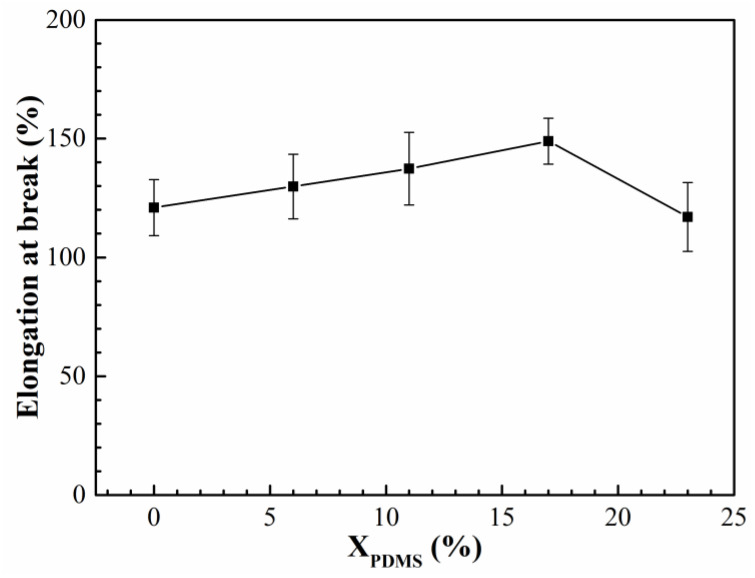
Elongation of modified silicone coatings.

**Figure 12:**
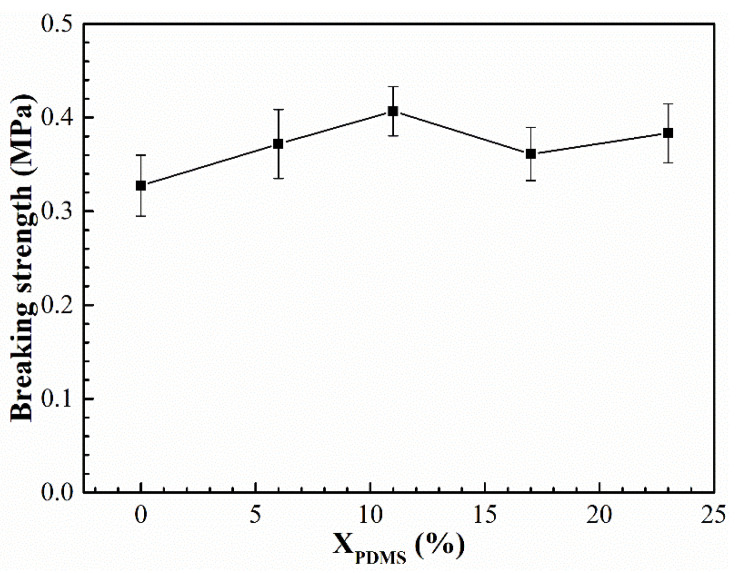
Breaking strength of modified silicone coatings.

The elongation and breaking strength of the modified coatings are shown in Figure 11 and Figure 12, respectively. It was found that with the increase of X_PDMS_, the elongation and breaking strength of the coatings both showed a trend of increasing first and then decreasing. This is because the addition of PDMS2200 improved the flexibility of the molecular chain, so its elongation was enhanced. At the same time, the hard segment generated by the microphase separation of the polyurethane acted as the second phase to strengthen the mechanical properties of the coating, thereby improving the breaking strength of the modified coatings; as X_PDMS_ continued to increase, the size of the hard segment was also growing, which made the deformation of the molecular chain more difficult, resulting in a decrease in the elongation of the coating. The increasing microphase separation led to an increase in the interface between soft and hard segments. These interfaces were more prone to stress concentration when subjected to external forces, which led to crack nucleation, so the fracture strength of the coating decreased.

### 3.5. Antifouling Performance

**Figure 13:**
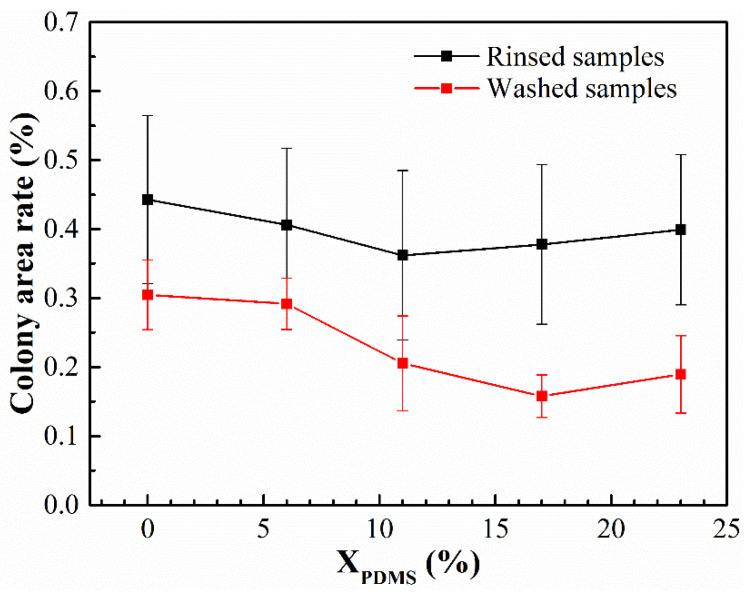
Colony area rate of the modified silicone coatings.

**Figure 14:**
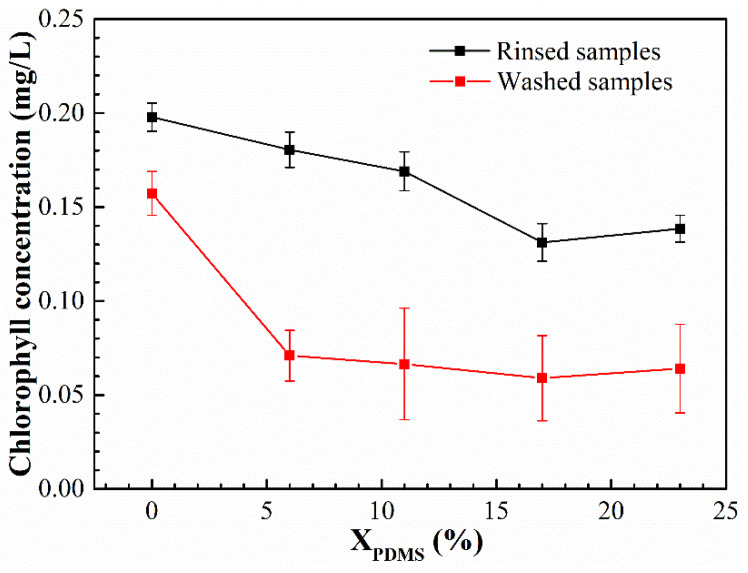
Chlorophyll concentration of the modified silicone coatings.

The bacterial and benthic diatom adhesion experiments were carried out on the modified coatings, and their colony area rate and chlorophyll concentration are shown in Figure 13 and Figure 14, respectively. It was found that with the continuous addition of PDMS2200, the colony area rate and chlorophyll concentration on the coatings’ surfaces showed a trend of first decreasing and then increasing. Furthermore, the washing treatment could effectively remove the bacteria and benthic diatoms attached to the coatings’ surfaces.

## 4. Discussion

**Figure 15:**
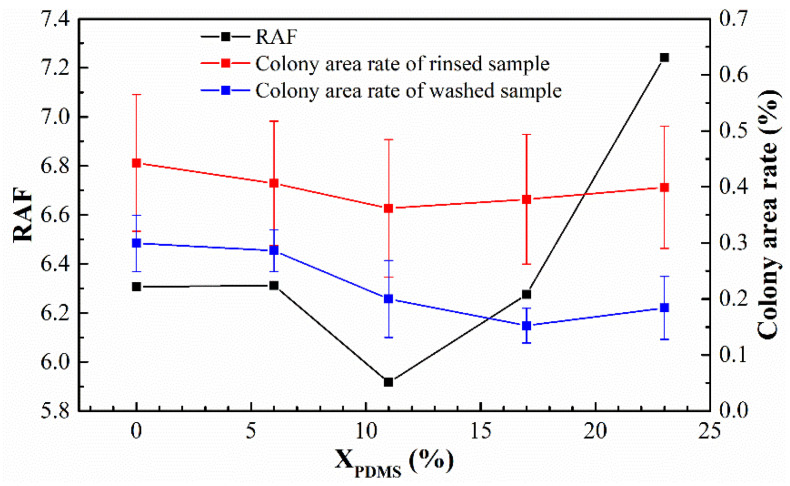
Relationship between the RAF and the colony area rate of the modified silicone coatings.

Brady [34] once proposed the relative adhesion factor (RAF), which is considered an essential basis for characterizing the antifouling performance of silicone coatings. RAF is calculated with the following equation:

$$\mathrm{RAF}=\sqrt{E\gamma }$$

where *E* denotes the elastic modulus of the coating, and *γ* denotes the surface free energy of the coating. The results of the RAF are shown in Figure 15. It was found that for the rinsed samples, the colony area rate was entirely in line with Brady’s theory, that is, the smaller the RAF, the better the antifouling performance of the coating; for the washed sample, the change of the colony area rate and RAF was not precisely the same, which may have been caused by the roughness *Ra* of the coating surface.

Therefore, based on RAF, the roughness of the coating surface was introduced, and a new relative adhesion factor RAF’ was proposed; it was calculated with the following equation:

$$\mathrm{RAF}’=\sqrt{E\gamma Ra}$$

**Figure 16:**
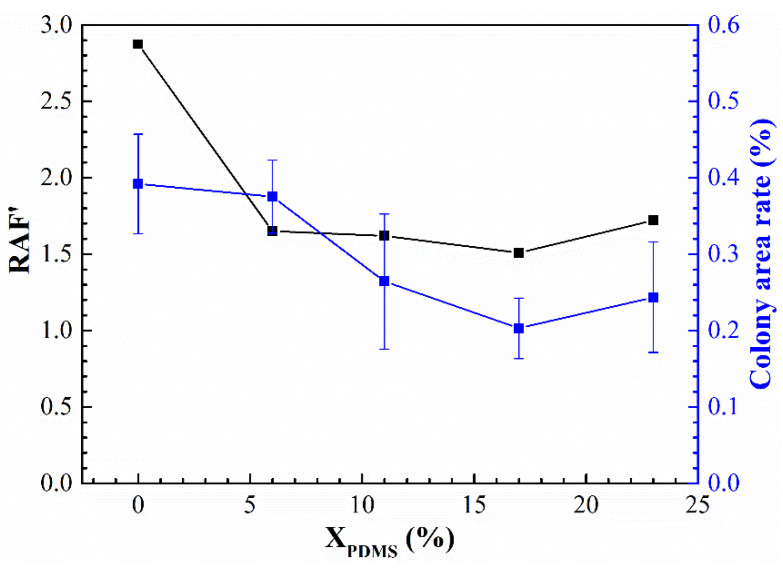
The relationship between the RAF’ and the colony area rate of the washed samples.

The results of RAF’ and the colony area rate of the washed samples are shown in Figure 16. It was found that the smaller the RAF’, the lower the colony area rate. This indicates that the removal of fouling organisms on the surface of the modified silicone coating prepared in this paper was controlled by its elastic modulus, surface energy, and roughness.

Based on the above, it can be seen that the increase of X_PDMS_ will aggravate the microphase separation of the modified coatings, thereby reducing the roughness of the coating surface, which will improve the wetting and dispersibility of the liquid on the coating surface to a certain extent. The introduction of PDMS2200 brings a large number of Si, as well as O atoms, which will improve the dispersion force of the modified coatings. Under the combined action of these two factors, the coating *γ* will continue to increase with the increase of X_PDMS_.

At the same time, the introduction of PDMS2200 will also increase the content of the soft segment, which will improve the deformation ability of the modified coatings, thereby reducing its E; when the content of PDMS2200 exceeds a certain level, the strengthening effect of the hard segment is reflected, and continuing to increase X_PDMS_ will lead to an increase in E instead of a decrease.

Therefore, by controlling X_PDMS_, the modified coatings can obtain a more suitable degree of microphase separation to obtain lower *E*, *γ*, and *Ra*, giving the modified coatings good antifouling performance.

## 5. Conclusions

In this paper, PED-TDI polyurethane-modified silicone coatings were prepared, and the effects of soft segments on the structure and properties of modified coatings were investigated by introducing different contents of PDMS2200 into polyurethane.

(1) By analyzing the infrared spectrum and NMR spectrum of PED-TDI polyurethane and its modified coatings, it was found that isocyanate-terminated polyurethane was successfully prepared, and it was successfully used to modify silicone;

(2) When X_PDMS_ = 23 mol%, the modified coating has the lowest surface roughness, which is 0.0565 μm; when X_PDMS_ = 0 mol%, the surface free energy of the modified coatings is the lowest, which is 26.01 mJ/m^2^; when X_PDMS_ = 11 mol%, the elastic modulus of the modified coatings is the lowest, which is 1.30 MPa;

(3) The antifouling effect of the modified silicone coatings is related to its own RAF’. Polyurethane with different soft segment compositions changes the coating’s elastic modulus, surface energy, and roughness by affecting the microphase separation degree, which finally influences the antifouling properties. When X_PDMS_ = 17 mol%, the modified coatings can obtain better mechanical properties and antifouling effects.

## Figures and Tables

**Table 1:** The raw material ratios of the soft segments.

	PU-Si0	PU-Si6	PU-Si11	PU-Si17	PU-Si23
PED (mol%)	89.89	85.01	79.73	74.66	69.82
PET (mol%)	10.11	9.40	8.88	8.37	7.66
PDMS2200 (mol%)	0	5.59	11.39	16.97	22.52

## References

[1] Xie Q., Pan J., Ma C., Zhang G. (2019). Dynamic surface antifouling: Mechanism and systems. Soft Matter.

[2] Martín-Rodríguez A.J., Babarro J., Lahoz F., Sansón M., Martin V., Norte M., Fernandez J.J. (2015). From Broad-Spectrum Biocides to Quorum Sensing Disruptors and Mussel Repellents: Antifouling Profile of Alkyl Triphenylphosphonium Salts. PLoS ONE.

[3] Leonardi A.K., Ober C.K. (2019). Polymer-Based Marine Antifouling and Fouling Release Surfaces: Strategies for Synthesis and Modification. Annu. Rev. Chem. Biomol. Eng..

[4] Hu P., Xie Q., Ma C., Zhang G. (2020). Silicone-Based Fouling-Release Coatings for Marine Antifouling. Langmuir.

[5] Liu C., Ma C., Xie Q., Zhang G. (2017). Self-repairing silicone coatings for marine anti-biofouling. J. Mater. Chem. A.

[6] Chambers L.D., Stokes K.R., Walsh F.C., Wood R.J.K. (2006). Modern approaches to marine antifouling coatings. Surf. Coat. Technol..

[7] Mirabedini S., Pazoki S., Esfandeh M., Mohseni M., Akbari Z. (2006). Comparison of drag characteristics of self-polishing co-polymers and silicone foul release coatings: A study of wettability and surface roughness. Prog. Org. Coat..

[8] Patwardhan S.V., Taori V.P., Hassan M., Agashe N.R., Franklin J.E., Beaucage G., Mark J.E., Clarson S.J. (2006). An investigation of the properties of poly(dimethylsiloxane)-bioinspired silica hybrids. Eur. Polym. J..

[9] Lejars M., Margaillan A., Bressy C. (2012). Fouling Release Coatings: A Nontoxic Alternative to Biocidal Antifouling Coatings. Chem. Rev..

[10] Wang L., Li L., Feng S. (2021). Research Progress of Silicone Supramolecular Materials. Chem. J. Chin. Univ. Chin..

[11] Yi B., Wang S., Hou C., Huang X., Cui J., Yao X. (2021). Dynamic siloxane materials: From molecular engineering to emerging applications. Chem. Eng. J..

[12] Han X., Wu J., Zhang X., Shi J., Wei J., Yang Y., Wu B., Feng Y. (2021). Special issue on advanced corrosion-resistance materials and emerging applications. The progress on antifouling organic coating: From biocide to biomimetic surface. J. Mater. Sci. Technol..

[13] Mejía M.C., Palacio J., Murillo E.A. (2017). Comb-shaped silicone-alkyd resins with high solid content. Prog. Org. Coat..

[14] Sun X., Chen R., Gao X., Liu Q., Liu J., Zhang H., Yu J., Liu P., Takahashi K., Wang J. (2019). Fabrication of epoxy modified polysiloxane with enhanced mechanical properties for marine antifouling application. Eur. Polym. J..

[15] Augustinho T.R., Motz G., Ihlow S., Machado R.A.F. (2016). Application of hybrid organic/inorganic polymers as coatings on metallic substrates. Mater. Res. Express.

[16] Szeto W., Leung M.K.H., Leung D.Y.C. (2021). Recent developments of titanium dioxide materials for aquatic antifouling application. J. Mar. Sci. Technol..

[17] Sui Z., Li Y., Guo Z., Zhang Q., Xu Y., Zhao X. (2022). Preparation and properties of polysiloxane modified fluorine-containing waterborne polyurethane emulsion. Prog. Org. Coat..

[18] Lewandowski K., Krepski L.R., Mickus D.E., Roberts R.R., Heilmann S.M., Larson W.K., Purgett M.D., Koecher S.D., Johnson S.A., McGurran D.J. (2002). Synthesis and properties of waterborne self-crosslinkable sulfo-urethane silanol dispersions. J. Polym. Sci. Pol. Chem..

[19] Xia J., Liu B., Yang M. (2018). Research Progress of Organosilicon Low Surface Energy Antifouling Coatings for Marine Vessels. Mater. Rev..

[20] Hua L., Xu H., Zheng F. (2016). Review of modified silicone coatings. Sci. Technol. Chem. Ind..

[21] Dai Z., Yang K., Dong Q. (2015). Synthesis and characterization of hydroxy-terminated polyether-polydimethylsiloxane-polyether (PE-PDMS-PE) triblock oligomers and their use in the preparation of thermoplastic polyurethanes. J. Appl. Polym. Sci..

[22] Barletta M., Aversa C., Pizzi E., Puopolo M., Vesco S. (2018). Design, manufacturing and testing of anti-fouling/foul-release (AF/FR) amphiphilic coatings. Prog. Org. Coat..

[23] Clarson S.J., Fitzgerald J.J., Owen M.J., Smith S.D. (2000). High Strength Silicone-Urethane Copolymers: Synthesis and Properties. Silicones Silicone-Modified Mater..

[24] Yang Z. (2018). Research on the Preparation and Properties of Polyurethane Elastomers Modified with Polydimethylsiloxane. Ph.D. Thesis.

[25] He Y., Xie D., Zhang X. (2014). The structure, microphase-separated morphology, and property of polyurethanes and polyureas. J. Mater. Sci..

[26] Wang C., Ma C., Mu C., Lin W. (2017). Tailor-made zwitterionic polyurethane coatings: Microstructure, mechanical property and their antimicrobial performance. RSC Adv..

[27] Zhang Q., Liu H., Zhan X., Chen F., Yan J., Tang H. (2015). Microstructure and antibacterial performance of functionalized polyurethane based on polysiloxane tethered cationic biocides. RSC Adv..

[28] Zhang Z.-P., Song X.-F., Cui L.-Y., Qi Y.-H. (2018). Synthesis of Polydimethylsiloxane-Modified Polyurethane and the Structure and Properties of Its Antifouling Coatings. Coatings.

[29] Oliver J.F., Mason S.G. (1980). Liquid Spreading on Rough Metal-Surfaces. J. Mater. Sci..

[30] Yang Q., Zhang Z., Qi Y., Zhang H. (2020). Influence of Phenylmethylsilicone Oil on Anti-Fouling and Drag-Reduction Performance of Silicone Composite Coatings. Coatings.

[31] Zhang Y., Qi Y.-H., Zhang Z.-P., Sun G.-Y. (2015). Synthesis of fluorinated acrylate polymer and preparation and properties of antifouling coating. J. Coat. Technol. Res..

[32] Cassie A.B.D., Baxter S. (1944). Wettability of porous surfaces. Trans. Faraday Soc..

[33] Gao N., Yan Y. (2009). Modeling Superhydrophobic Contact Angles and Wetting Transition. J. Bionic Eng..

[34] Brady R.F., Singer I.L. (2000). Mechanical factors favoring release from fouling release coatings. Biofouling.

